# Supplementary material for: Evidence supported by Mendelian randomization: impact on inflammatory factors in knee osteoarthritis
Source: Front Med (Lausanne). 2024 May 28;11:1382836. doi: 10.3389/fmed.2024.1382836 (PMC11165061; doi:10.3389/fmed.2024.1382836)
Supplement: Supplementary file 1 [file Data_Sheet_1.ZIP › data/GCST90274828_5e6/828.leaveoneout.pdf]

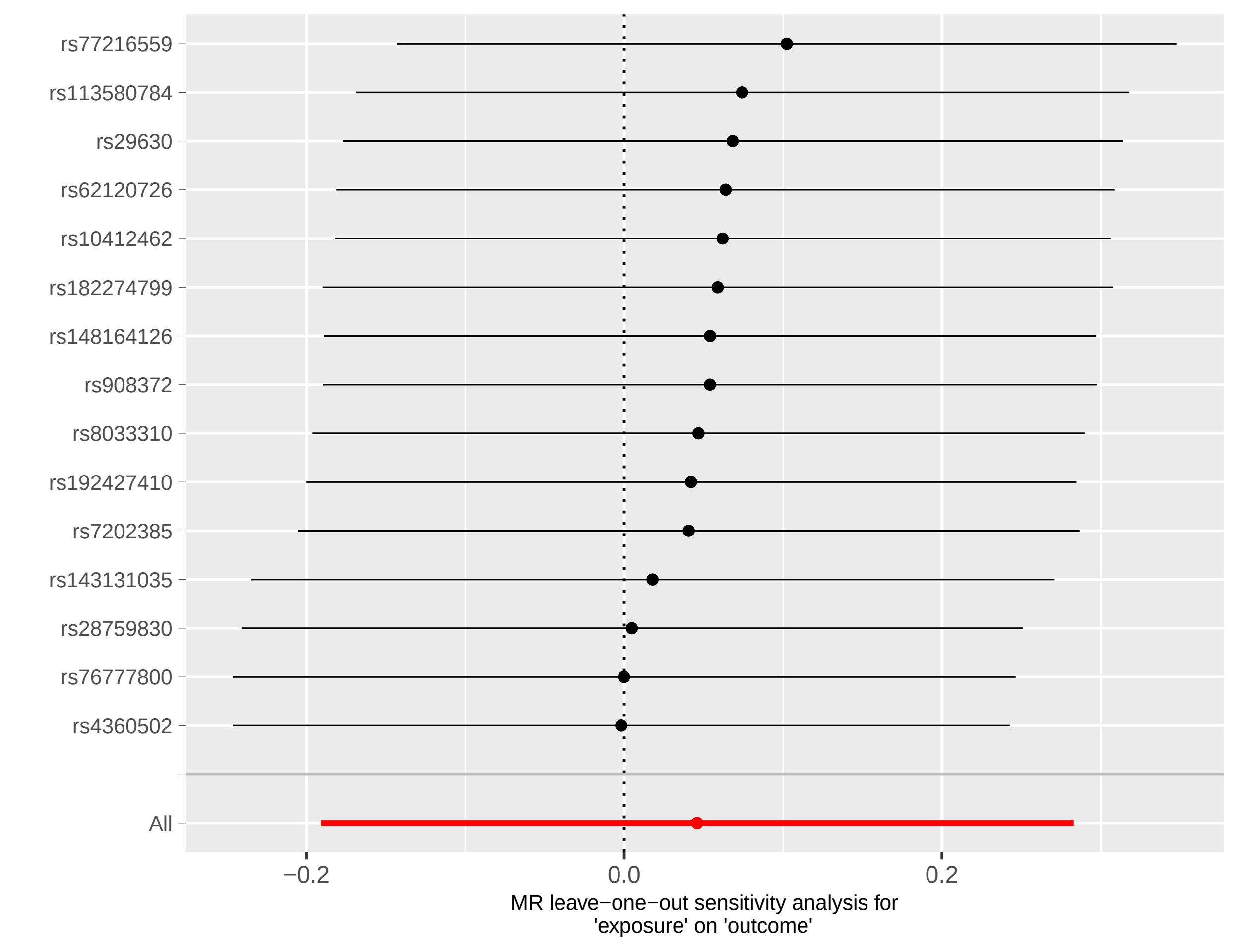
rs77216559
rs113580784
rs29630
rs62120726
rs10412462
rs182274799
rs148164126
rs908372
rs8033310
rs192427410
rs7202385
rs143131035
rs28759830
rs76777800
rs4360502
All
−0.2
0.0
0.2
MR leave−one−out sensitivity analysis for
'exposure' on 'outcome'
